# Characterization of the Bacterial Microbiome in Natural Populations of Barley Stem Gall Midge, *Mayetiola hordei*, in Morocco

**DOI:** 10.3390/microorganisms11030797

**Published:** 2023-03-21

**Authors:** Imane Remmal, Naima Bel Mokhtar, Amal Maurady, Mohammed Reda Britel, Karim El Fakhouri, Elias Asimakis, George Tsiamis, Panagiota Stathopoulou

**Affiliations:** 1 Laboratory of Innovative Technologies, National School of Applied Sciences of Tangier, Abdelmalek Essaâdi University, BP 1818 Tanger Principal, Tanger 90000, Morocco; 2 Faculty of Sciences and Technology of Tangier, Abdelmalek Essâadi University, Tétouan 93000, Morocco; 3 Laboratory of Systems Microbiology and Applied Genomics, Department of Sustainable Agriculture, University of Patras, 30100 Agrinio, Greece; 4 AgroBioSciences Program, College for Sustainable Agriculture and Environmental Science, Mohammed VI Polytechnic University, Lot 660, Hay Moulay Rachid, Ben Guerir 43150, Morocco

**Keywords:** barley stem gall midge, biological control, 16S rRNA gene, symbiosis, *Wolbachia*, next-generation sequencing (NGS)

## Abstract

**Simple Summary:**

Barley stem gall midge, *Mayetiola hordei* (Kieffer), is one of the most devastating barley pests in many regions of the world, causing significant losses to agricultural output. In this work, we explore the presence of reproductive symbionts, including *Wolbachia*, *Spiroplasma*, *Cardinium*, and *Arsenophonus*, which can be employed as a substitute for chemical pesticides in pest management, in addition to the impact of their geographic origin on bacterial composition, as well as the diversity of the natural populations of barley stem gall midges detected in four Moroccan barley-growing regions. The screening revealed the presence of *Wolbachia*; in contrast, none of the samples analyzed had *Spiroplasma*, *Cardinium*, or *Arsenophonus* infections. Overall, 5 phyla, 7 classes, and 42 genera were detected throughout all samples, with a significant environmental influence on the taxonomic assortment of the microbiota.

**Abstract:**

*Mayetiola hordei* (Kieffer), known as barley stem gall midge, is one of the most destructive barley pests in many areas around the world, inflicting significant qualitative and quantitative damage to crop production. In this study, we investigate the presence of reproductive symbionts, the effect of geographical origin on the bacterial microbiome’s structure, and the diversity associated with natural populations of *M. hordei* located in four barley-producing areas in Morocco. *Wolbachia* infection was discovered in 9% of the natural populations using a precise 16S rDNA PCR assay. High-throughput sequencing of the V3-V4 region of the bacterial 16S rRNA gene indicated that the native environments of samples had a substantial environmental impact on the microbiota taxonomic assortment. Briefly, 5 phyla, 7 classes, and 42 genera were identified across all the samples. To our knowledge, this is the first report on the bacterial composition of *M. hordei* natural populations. The presence of *Wolbachia* infection may assist in the diagnosis of ideal natural populations, providing a new insight into the employment of *Wolbachia* in the control of barley midge populations, in the context of the sterile insect technique or other biological control methods.

## 1. Introduction

*Mayetiola hordei* (Kieffer) (Diptera: Cecidomyiidae), the barley stem gall midge (BSGM), is a small fly (2–4 mm in length), and one of the most devastating pests of cereal crops. This insect is native to the Mediterranean regions in North Africa, including Morocco, Algeria, and Tunisia, and additionally to Southern Europe, Spain, and Italy [1]. Infestation rates and yield losses can become severe after BSGM is established in a region or country. For example, around 30–50% of barley crops in Libya and Tunisia are infested by this pest and cereal production in Morocco is decreased by about 35% due to the damage caused by *M. hordei* [2,3]. This insect has a long-lived larval stage with two feeding stages and a third non-feeding stage, during a generation time that is completed in 45 days at an average temperature of (18 ± 1 °C) [3]. Adverse effects on crops occur during this developmental stage. The principal symptom in seedlings is a yellowing of new growth, which can occasionally lead to plant mortality. BSGM puparia are always embedded in galls and associated with plant stunting [2,4]. The female can lay up to 400 [5]. The most practical approach to manage BSGM is prevention, including insecticide applications and planting adjustment, while the use of resistant cultivars is not common, although it is considered one of the most successful and cost-efficient strategies to control these insects [6,7]. 

Among the methods that can fulfill the projected increased need for novel insect pest management techniques brought on by expanding human populations and climate change are those based on symbiotic microorganisms [8]. Insects coexist in a variety of ways with many different microorganisms [9,10]. Numerous bacterial species and insect hosts have been shown to form complex symbiotic relationships that can be classified as commensalism, mutualism, or parasitism, which affect a variety of elements of the host’s biology, physiology, ecology, and evolution, including nutrition, reproduction, mating behavior, fitness, immunity, and status as a pest [11,12,13]. The interactions between insects and their endosymbionts, especially those related to mechanisms of insect reproduction, are considered as a rapid, environmentally friendly, and efficient biological approach to suppress or eliminate insect infestations [8]. In this way, insects that damage crops and spread diseases to people, plants, and domestic animals have been managed, and in many cases eliminated, resulting in improved standards of living and quality of life throughout the world [14]. *Wolbachia* is an α-proteobacterium, an intracellular symbiont discovered in 1924 [15], present in a wide variety of arthropod taxa, including insects, mites, spiders, springtails, crustaceans, and some nematodes. More than 20% of arthropod species may be *Wolbachia*-infected, according to several PCR-based assessments. Based on their main genetic changes across a variety of hosts, *Wolbachia* have recently been grouped into 17 supergroups, represented by the letters A to S [16,17,18]. Supergroup G, which was recently eliminated because it was likely a combination of supergroups A and B [19,20], and the cave spider supergroup R, which showed a strong affinity for supergroup A, are exceptions [21,22]. *Wolbachia* can infect and maintain itself in hosts by manipulating their reproduction [23,24]. *Wolbachia*-induced cytoplasmic incompatibility (CI) is one of several techniques that can be differentiated and advantageous in two ways: as a method for controlling insect pest populations, similar to the sterile insect technique, and as a drive system for spreading genotypes in host populations. Furthermore, pathogenic *Wolbachia* strains also have the ability to manage vector species by altering the age structure of their populations [9,25]. *Wolbachia* also plays an important role in aspects of its host ecology, such as reproduction, nutrition, pathogen resistance, and fecundity [26].

In addition to *Wolbachia,* other endosymbiotic bacteria that can affect the biology of their insect hosts exist, such as for *Spiroplasma*, an extracellular symbiont belonging to the class Mollicutes, which can kill males in a variety of insect hosts, including *Drosophila* flies, butterflies, and lady beetles. Moreover, these bacteria can confer protection to the infected host against natural adversaries [27,28]. The endosymbiont *Cardinium* belonging to the Bacteroidetes is also known to be involved in arthropod reproduction manipulation, causing cytoplasmic incompatibility, parthenogenesis, and feminization to its hosts [29,30]. Furthermore, the bacterial symbiont *Arsenophonus* can induce a male-killing phenotype, resulting in high-rate mortality of male embryos produced by infected females. The bacterium has no discernible effect on the host. Therefore, it can propagate throughout the ecological community of its hosts, such as filth flies and their parasitoids, colonize new species, and remain in the population for lengthy periods of time [31,32].

To the best of our knowledge, this is the first study about the BSGM bacterial microbiome, although a few studies have recently been published on the characterization of the bacterial community associated with the Moroccan Hessian fly (*Mayetiola destructor*), an insect that shares a similar distribution to BSGM, similar morphology, and comparable symptoms concerning host plants [33]. According to Bel Mokhtar et al. (2020), the sequencing of the 16S rRNA bacterial gene (V3-V4 region) revealed various taxa at the phylum level, including Proteobacteria, Bacteroidetes, Actinobacteria, Cyanobacteria, Deinococcus-Thermus, and Firmicutes. At the genus level, different frequencies were observed between collection regions in Morocco for *Empedobacter*, *Ralstonia*, *Afipia*, and *Pseudomonas*. Regarding *Wolbachia*-infection, it was detected in all Moroccan regions with a different relative abundance [34]. Additionally, Bansal et al. (2011) identified four phyla in Hessian fly adults based on classical sequencing: Proteobacteria, Firmicutes, Actinobacteria, and Bacteroidetes. At the genus level, various types of bacteria were detected, including *Enterobacter*, *Pantoea*, *Stenotrophomonas*, *Pseudomonas*, *Bacillus*, *Ochrobactrum*, *Acinetobacter*, *Alcaligenes*, *Nitrosomonas*, *Arcanobacterium*, *Microbacterium*, *Paenibacillus*, and *Klebsiella* [35].

The aim of this study is to screen natural populations of BSGM midge for the presence of reproductive symbionts, *Wolbachia*, *Spiroplasma*, *Arsenophonus*, and *Cardinium,* using the 16S rRNA gene. In addition, a high-throughput sequencing (HTS) approach is performed using the Illumina-MiSeq technology to investigate the bacterial symbionts associated with natural populations of BSGM larvae to elucidate the relationship between the insects’ bacterial community profile and their habitat.

## 2. Materials and Methods

### 2.1. Barley Stem Gall Midge Collection and DNA Isolation

Plants infected with BSGM were obtained from four major barley-producing areas in Morocco: Abda, Rabat-Salé-Zemmour-Zaër, Fès-Meknes, and Doukkala. All infested plant samples were kept at the International Center of Agricultural Research in Dry Areas (ICARDA) in Rabat, Morocco (Table 1), where the BSGM larvae and pupae were collected during the winter of 2020. Larvae were stored in absolute ethanol at −20 °C, whereas pupae protected in infested plant tissues were reared in a growth room under constant conditions (temperature 23 ± 2 °C, relative humidity 65 ± 5%). Once the adults emerged, they were stored in 100% ethanol at −20 °C. Before DNA extraction, each sample was surface sterilized with a 70% *v*/*v* ethanol solution, rinsed with sterile deionized water to remove traces of ethanol, left to dry on a sterile surface, and placed in 1.5 mL centrifuge tubes. After surface sterilization, whole-fly DNA was isolated using a modified CTAB (cetyl trimethyl ammonium bromide) method [36]. The quantity and quality of the DNA preparations and the concentration of double-stranded DNA were both analyzed by a Q5000 micro-volume UV-Vis spectrophotometer (Quawell Technology, San Jose, CA, USA). DNA samples were stored in Eppendorf tubes at −20 °C until further analysis.

### 2.2. Screening and Identification of Bacterial Symbionts 

From the BSGM samples collected, 152 insects were selected (Table 1), including 108 that were acquired from 4 different areas, whereas only 2 regions (Rabat and Fez Meknes) were the source of the 44 adult specimens. However, the larvae from Doukkala and Abda were unable to develop into adults in the laboratory setting. The detection of *Wolbachia, Spiroplasma, Cardinium*, and *Arsenophonus* was performed by PCR assays using 16S rDNA genus specific primers. Blank and negative controls were incorporated during DNA extractions, and the PCRs were conducted using identical conditions. However, no amplicons were produced from these samples. The quality of the extracted DNA was tested by amplifying part of the mitochondrial 12S rRNA gene (Table S2). Amplification was performed in 25 µL reaction mixtures containing 2.5 µL KAPA Taq buffer (KAPA BioSystems, Wilmington, MA, USA) 10×, 0.25 µL dNTPs (25 mM), 0.25 µL of KAPA Taq DNA polymerase, 0.5 µL of the forward primer (25 µM), 0.5 µL of the reverse primer (25 µM), 1 µL of template DNA solution, and was finalized with 20 µL sterile deionized water. The PCR temperature profile was 95 °C for 5 min followed by 35 cycles of 95 °C for 30 s, 30 s at the optimum annealing temperature for each pair of primers, 1 min at 72 °C, and a final extension step of 72 °C for 5 min. PCR products were electrophoresed on a 1.5% agarose gel in order to examine the presence and size of the amplified fragments. The primer sequences used in this study along with the product size and annealing temperature are summarized in Table S2. The PCR-positive products were purified using polyethylene glycol (20% PEG, 2.5 M NaCl) [37] and resuspended in 15 µL of water. Sanger sequencing was performed on purified products with the BigDye Terminator v3.1 Cycle Sequencing Kit following the manufacturer’s recommendations (Applied Biosystems, Waltham, MA, USA). Reaction products were purified using an ethanol/EDTA protocol according to the manufacturer’s instructions (Applied Biosystems, Waltham, MA, USA) and were analyzed in an ABI PRISM 3500 Genetic Analyzer (Applied Biosystems, Waltham, MA, USA). 

### 2.3. Amplification of the V3-V4 Region, Index PCR, Purification, and Illumina Sequencing

For the high-throughput amplicon sequencing analysis, 78 samples of larvae were selected. However, 3 of these larvae (1 from Abda, 1 from Doukkala, and 1 from Rabat) were disregarded because they had low read counts (less than 1000) to prevent issues related to infinite values that may arise when applying the logarithmic transformation. Therefore, the final number of larvae analyzed was 75, including 19 larvae from Rabat, 17 from Abda, 19 from Doukkala, and 20 from Fes-Meknes (Table 1). The hypervariable V3-V4 region of the bacterial 16S rRNA gene was amplified using MiSeq universal primers 341F and 805R (Table S2). The PCR amplification was performed in two steps. The first amplification was performed using the bacterial universal primers 27F-1492R (Table S2) in a 20 μL reaction comprising 2 μL of KAPA Taq buffer 10×, 0.16 μL of dNTPs (25 mM), 0.16 μL of 200 KAPA Taq DNA polymerase (Roche, Basel, Switzerland), 0.4 μL of forward primer (25 μM), 0.4 μL of reverse primer (25 μM), 1 μL of the template DNA solution, and 15.88 μL of water. A 3 min incubation time at 95 °C was used for DNA denaturation, followed by 20 cycles of 95 °C for 30 s, 53 °C for 30 s, and 72 °C for 2 min, with a final 5 min extension at 72 °C. The second amplification included a nested PCR using MiSeq universal primers 341F and 805R. The amplification was performed in 25 µL reaction mixtures containing 2.5 µL KAPA Taq buffer 10×, 0.2 µL dNTPs (25 mM), 0.1 µL of KAPA Taq DNA polymerase, 1 µL of the forward primer (25 µM), 1 µL of the reverse primer (25 µM), 1 µL of the first-step reaction as template, and was finalized with 19.2 µL of sterile deionized water. The PCR amplifications were performed with incubation at 95 °C for 3 min, followed by 21 cycles of 95 °C for 30 s, 54 °C for 30 s and 72 °C for 1 min, and a final 5 min extension at 72 °C. For the Illumina sequencing, the resulting PCR amplicons were used as templates for further amplification, to include the indexes (barcodes) as well as the Illumina adaptors, in a 50 µL volume containing 5 µL KAPA Taq buffer 10×, 0.4 µL dNTPs (25 mM), 0.2 µL of KAPA Taq DNA polymerase, 5 µL of the forward index primer (10 µM), 5 µL of the reverse index primer (10 µM), 2 µL of the cleaned PCR product diluted up to 10 ng/µL, and 32.4 µL of sterile deionized water. The PCR amplifications were performed with incubation at 95 °C for 3 min followed by 8 cycles of 95 °C for 30 s, 30 s at 55 °C, 30 s at 72 °C, and a final extension step of 72 °C for 3 min. The resulting amplicons from the indexing PCR were cleaned using the NucleoMag NGS (next-generation sequencing) clean-up and size selection kit (Macherey-Nagel, Düren, Germany), according to the manufacturer’s recommendations. From all samples examined, the indexed amplicons were mixed in an equimolar ratio (8 nM) and the sequencing was performed by Macrogen using a 2 × 300 bp pair-end kit on a MiSeq platform [38]. Raw sequencing reads were demultiplexed, converted to FASTQ, and the Illumina adapters were trimmed using Illumina standard algorithms.

### 2.4. NGS Sequencing and Bioinformatics Analysis

Bioinformatics analysis of raw sequencing reads was performed using USEARCH v. 11 and QIIME2 distribution [39,40]. Paired-end reads were assembled and trimmed by length using the usearch -fastq_mergepairs command. The quality of assembled sequences was improved using -fastq_filter, followed by the -fastx_uniques command to detect unique read sequences and their frequencies. Sequences were clustered into operational taxonomic units (OTUs) using the -cluster_otus command at 90% OTU clustering based on the UPARSE algorithm [41]. Crosstalk errors were identified and filtered using the -uncross command based on the UNCROSS2 algorithm [42]. The taxonomy was assigned with Qiime2 using the BLAST + algorithm against the SILVA 138 release database [43,44]. The diversity of individual samples was identified by calculating alpha diversity metrics (evenness, Richness, Shannon, and Simpson indexes) using the “diversity” function of the R package “vegan”. Alpha diversity indexes were plotted with the ggplot function from the “ggplot2” package. Statistical differences in bacterial compositions between populations were tested using the nonparametric Kruskal and Wallis and Wilcoxon rank-sum tests [45]. To assess the similarity of bacterial communities in different locations, a beta diversity analysis was performed using the generalized UniFrac distance and visualized by non-metric multidimensional scale (NMDS) maps [46]. Permutational multivariate analysis of variance (PERMANOVA) using distance matrices was performed using the “adonis” function from the R package “vegan” to identify significant differences between isolated groups. A *p*-value < 0.05 was considered indicative of statistical significance.

A network analysis of OTUs was performed to investigate and visualize the interactions between microorganisms, which could be explained by similar or complementary functions and/or sharing similar environmental conditions, but not necessarily having physical interactions [47,48]. Networks were obtained using the CoNet plugin in Cytoscape 3.8.2 (Institute for System Biology, Seattle, WA, USA) and visualized with Gephi 0.9.2 (Institute for System Biology, Seattle, WA, USA) (Gephi, WebAtlas, Paris, France) [49]. The network was built using Pearson’s and Spearman’s rank correlation coefficients, mutual information, and the Bray–Curtis and Kullback–Leibler dissimilarity indices. The statistical significance of copresence/mutual exclusion, edge-specific permutation, and bootstrap score distributions were calculated using 1000 iterations. Edges with original scores outside the 0.95 range of their bootstrap distribution were rejected, and *p*-values were corrected using the Benjamini–Hochberg method.

### 2.5. Phylogenetic Analysis 

Phylogenetic analysis was based on partial 16S rRNA gene sequences obtained from *Wolbachia*-infected specimens and sequences representing *Wolbachia*-associated OTUs obtained by Illumina MiSeq sequencing. Multiple alignments were performed with MUSCLE, implemented in MEGA 7 software, using standard algorithm parameters [50,51]. Alignments were manually edited and trimmed to adjust the sequence length. Phylogenetic tree reconstruction was based on the maximum likelihood statistical model and was performed using MEGA 7 software. The (GTR + G + I) substitution model was used to estimate nucleotide evolution [52,53]. A bootstrap test with 1000 iterations was used to assess the reliability of the produced phylogeny [54]. The entirety of the 16S rRNA gene sequences produced in this work were submitted to the GenBank database of NCBI with accession numbers OQ189903-OQ189915.

### 2.6. Core Microbiome

Core microbiome analyses were performed using microbiome R package based on the *M. hordei* dataset [55]. The analysis was performed to detect major bacterial taxa that were recognized in 75% of the samples with a relative abundance threshold value greater than 0.01%, as well as to determine the taxa that were common between the different population regions, and to differentiate them from those which were specific to some regions.

## 3. Results

### 3.1. Infection Status of Reproductive Symbionts in Natural Populations of BSGM

#### 3.1.1. Infection Prevalence 

**Figure 1:**
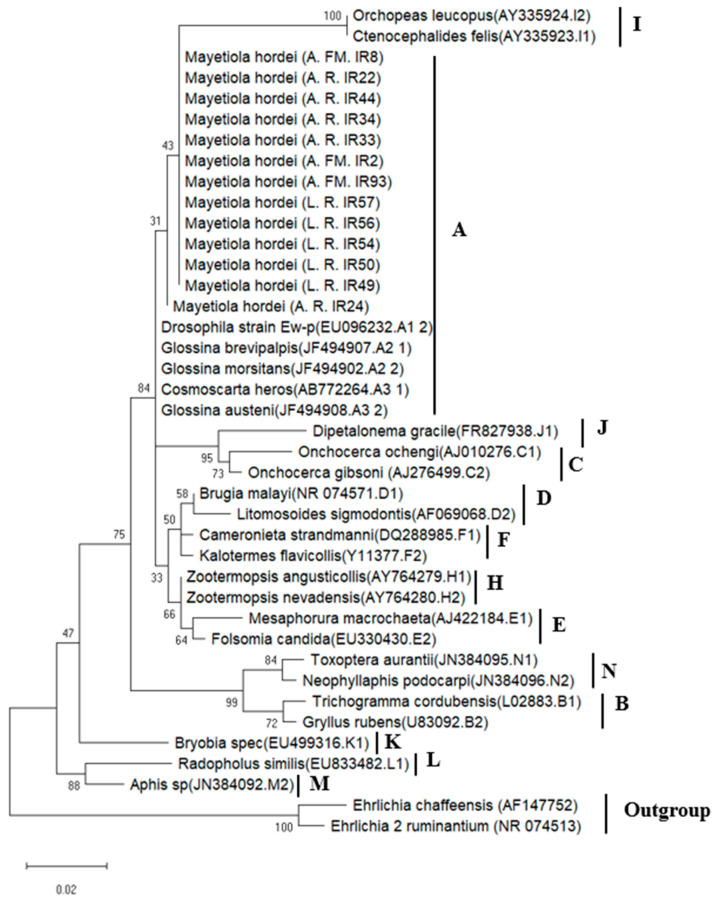
Maximum likelihood phylogenetic tree of the 13 Wolbachia strains found in barley stem gall midges based on 16S rRNA gene sequences (325 bp) (A: adult, L: larva, R: Rabat, FM: Fes-Meknes). Host species and the GenBank accession numbers are indicated for sequences of representatives of the Wolbachia supergroups A−N. Numbers in each node are bootstrap proportions based on 1000 replications (only values higher than 30% are shown).

PCR screening was used to assay the presence of four reproductive symbionts (*Wolbachia*, *Spiroplasma*, *Cardinium*, and *Arsenophonus*) in four natural BSGM populations. In total, 152 BSGMs were screened for reproductive symbionts (Table S1). The screening results indicate that the barley midge is infected only by *Wolbachia* with a prevalence of 9%. Interestingly, the *Wolbachia* percentage infection in natural populations did not reveal an even distribution among the different locations. In fact, only BSGMs from Rabat and Fes-Meknes were found to be infected with *Wolbachia*. In total, 13 flies were infected, five larvae, two females, and three males, out of 44 samples examined from Rabat (22%), and one larva, one female, and one male, out of 52 samples examined from Fes-Meknes (6%) (Table S1, Figure 1). On the contrary, none of the BSGM populations examined were infected with *Spiroplasma*, *Cardinium*, or *Arsenophonus*.

#### 3.1.2. Phylogenetic Analysis of *Wolbachia* Sequences in BSGM Populations

The *Wolbachia* phylogenetic analysis was performed on the thirteen *Wolbachia*-infected samples based on the partial 16S rRNA gene sequences, using a total of 325 bp (File S1) remaining in the thirteen *Wolbachia* sequences after manually removing the low-quality bases. The 325 bp still contained enough informative data to establish a distinct phylogenetic signal for *Wolbachia* found in insects, thus allowing an accurate inference of their evolutionary relationships [56,57,58]. According to the results, the *Wolbachia* strains detected in BSGM populations belonged to supergroup A, exhibiting a high sequence similarity of pairwise distances (98.5%) with *Wolbachia* sequences isolated from *Glossina* and *Drosophila* species (Figure 1).

### 3.2. 16S rRNA Amplicon Sequencing 

Applying Illumina high-throughput sequencing of 16S rRNA gene amplicons, the bacterial community composition and diversity of 75 natural barley stem gall midge samples from Rabat, Doukkala, Fes-Meknes, and Abda locations were examined. After sequencing and quality filtering, a total of 1,757,130 qualified paired-end reads with an average count per sample of 44,504 reads were divided into 638 OTUs. Based on a 90% sequence similarity, 61 OTUs were classified in five phyla, that is, for Proteobacteria, Firmicutes, Dependentiae (a recently characterized phylum, Table S3), Bacteroidetes, and Actinobacteria. Seven classes, Gammaproteobacteria, Alphaproteobacteria, Bacilli, Bacterodia, Actinobacteria, Negativicutes (a new class, Table S4), and Babeliae, plus 42 genera were procured across all the samples (Table S5). 

#### 3.2.1. Bacterial Diversity among BSGM Natural Populations

**Figure 2:**
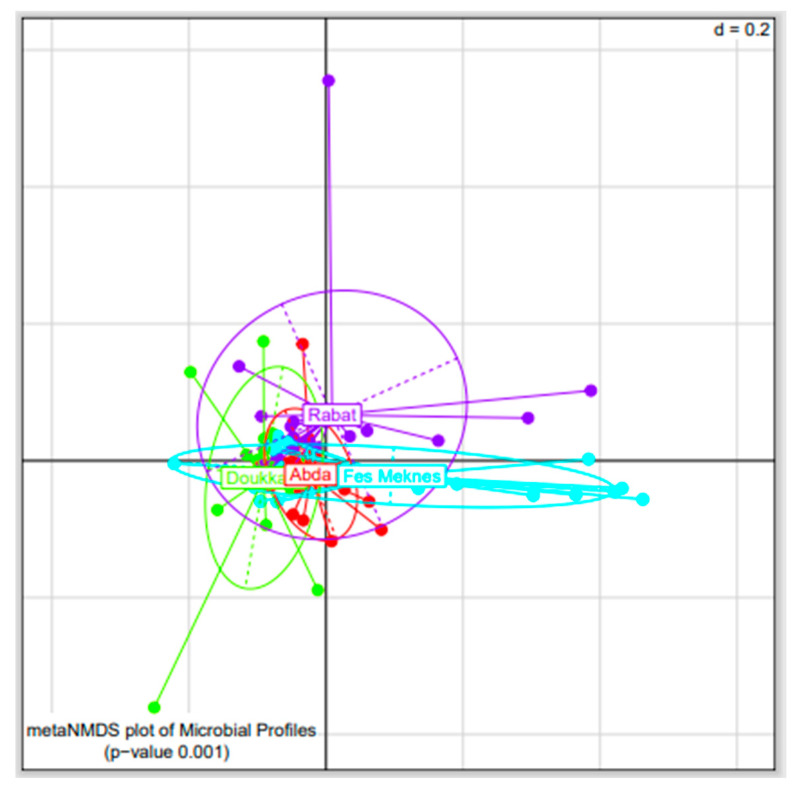
Nonmetric multidimensional scaling (NMDS) plot of bacterial communities in barley stem gall midge samples collected from Rabat (purple), Fes-Meknes (blue), Abda (red), and Doukkala (green) (*p* < 0.001).’d’ stands for the grid dissimilarity scale (d = 0.2 implies that the distance between two grid lines represents approximately 20% dissimilarity between the regions).

The bacterial communities of BSGM were clustered according to their geographic origins. The NMDS plots based on the generalized UniFrac distance revealed that samples from Rabat, Fes-Meknes, Abda, and Doukkala were significantly separated (PERMANOVA, *p* < 0.001, Figure 2), with similar results also obtained with pairwise analyses (Supplementary Figure S1).

Based on the number and relative abundance of OTUs and Simpson and Shannon indices (Figure S2), the four natural populations of BSGM were characterized by different species’ richness and diversity. The samples from Abda, Doukkala, and Fes-Meknes contained most of the bacterial species. Rabat samples, on the other hand, had statistically lower species richness and diversity than the other locations. Furthermore, based on the Shannon score, samples from the Doukkala region had statistically higher diversity than samples from the Rabat region (pairwise ANOVA: *p* < 0.05).

#### 3.2.2. Bacterial Composition of BSGM Natural Populations

**Figure 3:**
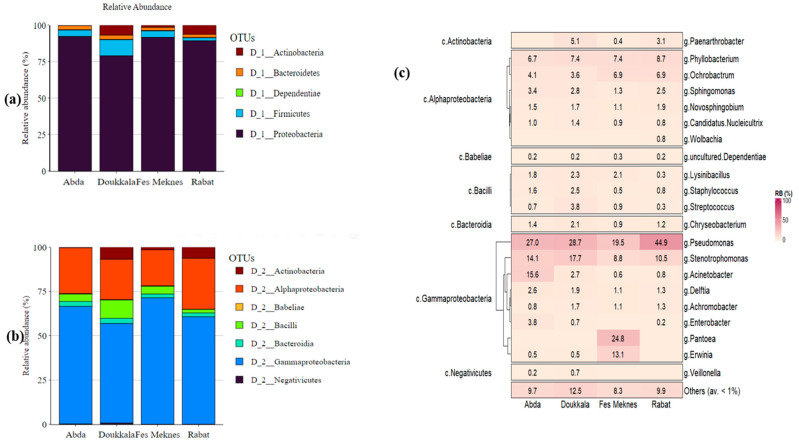
Relative abundance of natural barley stem gall midge populations’ microbiota at the phylum (**a**), class level (**b**), and heat map (**c**) of bacterial genera and classes identified in barley stem gall midge populations from the regions of Rabat, Fes-Meknes, Abda, and Doukkala.

The most abundant phylum detected in the four regions of Abda, Doukkala, Fes-Meknes, and Rabat was Proteobacteria (92.33 ± 1.01%, 79.05 ± 4.61%, 91.75 ± 1.8%, 89.41 ± 2.75%, respectively), while the classification of the rest of the phyla was different from one region to another. In Abda and Fes-Meknes, the second phylum was Firmicutes (4.34 ± 1.08%, 4.43 ± 1.4%, respectively), followed by Bacteroidetes (2.78 ± 0.42%, 2.08 ± 0.58%), Actinobacteria (0.31 ± 0.1%, 1.48 ± 0.75%), and Dependentiae (0.24 ± 0.1%, 0.26 ± 0.13%). In Doukkala, the second phylum was Firmicutes (11.00 ± 3.79%), followed by Actinobacteria (6.80 ± 3.58%), Bacteroidetes (2.94 ± 0.46%), and Dependentiae (0.21 ± 0.09%), while in Rabat, the second phylum was Actinobacteria (6.26 ± 2.83%), followed by Bacteroidetes (2.18 ± 0.4%), Firmicutes (2 ± 0.69%), and Dependentiae (0.15 ± 0.1%) (Figure 3a). The evaluation at the class level revealed that Gammaproteobacteria and Alphaproteobacteria were the most dominant members in all samples examined, followed by Bacilli in Abda, Doukkala, and Fes-Meknes. By contrast, in Rabat, the third class was Actinobacteria. Interestingly, members of Negativicutes and Babeliae were also detected, but to a much smaller degree (Figure 3b). At the genus level, the most abundant sequences in Abda, Doukkala, and Rabat were affiliated with *Pseudomonas* (27.02 ± 2.25%, 28.67 ± 2.44%, 45.24 ± 5.55%, respectively), followed by *Stenotrophomonas* (14.15 ± 1.44%, 17.68 ± 2.17%, 10.61 ± 1.42%, respectively), while the individuals from Fes-Meknes had a high relative abundance of *Pantoea* (24.80 ± 7.79%), followed by *Pseudomonas* (19.51 ± 3.34%). Different frequencies were observed for the remaining genera between the collection regions. In Abda, the third most prevalent OTU was *Acinetobacter*, followed by *Phyllobacterium* and *Ochrobactrum* (15.59 ± 3.53%, 6.72 ± 1.16%, 4.14 ± 0.84%, respectively); in Doukkala, the third abundant OTU was *Phyllobacterium*, followed by *Paenarthrobacter* and *Streptococcus* (7.37 ± 1.12%, 5.15 ± 3.08%, 3.85 ± 2.85%, respectively); in Fes-Meknes, the third genus was *Stenotrophomonas*, followed by *Phyllobacterium* and *Ochrobactrum* (8.78 ± 1.57%, 7.41 ± 1.32%, 6.92 ± 1.42%, respectively); and in Rabat, the third OTU was *Phyllobacterium*, followed by *Ochrobactrum* and *Allorhizobium-Neorhizobium-Pararhizobium-Rhizobium* (8.78 ± 1.18%, 6.93 ± 1.26%, 3.70 ± 2.98%, respectively). The frequencies of the rest of the genera were less than 0.1 (Figure 3c). *Wolbachia* was detected in samples from Rabat and Abda (Table S6) with a low relative abundance (0.75 ± 0.48%, 0.001 ± 0%, respectively). The absence of *Spiroplasma, Cardinium*, and *Arsenophonus* in the bacterial microbiome of BSGM populations was also confirmed by amplicon sequencing data.

#### 3.2.3. *Wolbachia* Phylogenetic Analysis 

**Figure 4:**
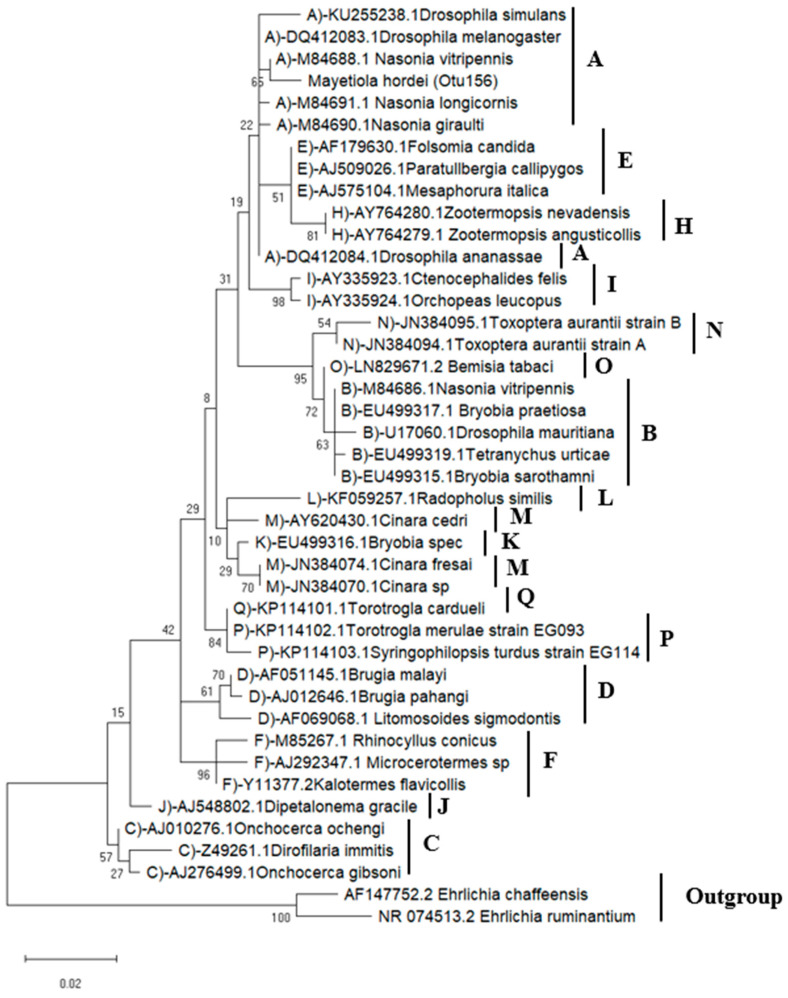
Maximum likelihood phylogenetic tree based on *Wolbachia*-related OTUs (16S rRNA gene) (443 bp full-size alignment): *Wolbachia*-related sequence acquired from positive Barley stem gall midge samples, as are the other sequences representing the known supergroups from A to Q. The names of the host species and GenBank accession numbers are used to identify *Wolbachia* sequences. Bootstrap proportions based on 1000 replication are shown by the number in each node.

Using conventional sequence clustering into OTUs, the *Wolbachia* infection status was determined from normalized libraries (90% sequence identity). *Wolbachia*-related reads were clustered into a single OTU (OTU 156), with prevalence discrepancies between the two regions investigated. In Rabat, seven samples out of 20 were infected, whereas, in Abda, one sample out of 18 was found to be infected with *Wolbachia*. The sequence of the *Wolbachia*-related OTU (443 bp) was positioned within the supergroup A sequences according to the phylogenetic analyses based on the 16S rRNA gene (Figure 4). These results indicate that the BSMG derived from different regions most likely carries the same *Wolbachia* strain.

#### 3.2.4. Bacterial Co-Occurrence/Mutual Exclusion Network Analysis of BSGM Natural Populations

**Figure 5:**
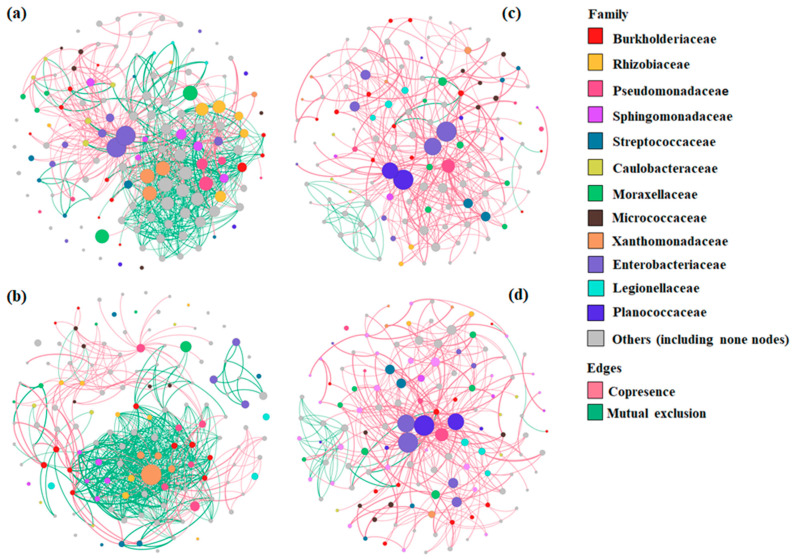
Co-occurrence and mutual exclusion networks at the family level for OTUs that compose the bacterial communities of *Mayetiola hordei* natural populations from the four regions: Fes-Meknes (**a**), Rabat (**b**), Doukkala (**c**), and Abda (**d**). The degree of interaction determines the size of each node. Cases of copresence are represented by green edges, while mutual exclusion is represented by red edges.

Co-occurrence and mutual exclusion network analysis was performed to examine the potential interactions between bacterial partners in each region separately. The networks for each region were shown at the family (Figure 5) and genus (Figure S3) levels. The number of OTUs (nodes), interactions (edges), and clustering coefficients varied amongst the four regions. The Fes-Meknes region had the highest number of nodes (144), followed by Rabat, Doukkala, and Abda (137, 132, and 122, respectively). Bacterial communities from Fes-Meknes had more interactions (1179) and a lower clustering coefficient (0.135) than Rabat (1177 edges and 0.215 coefficient), Doukkala, and Abda (395 edges and 0.215 coefficient, 350 edges and 0.041 coefficient, respectively). At the family level, mutual exclusions accounted for the majority of interactions in Doukkala and Abda (91.39% and 75.31%, respectively) (Figure 5c,d), while in the two left regions, Fes-Meknes and Rabat (Figure 5a,b), copresence accounted for the majority of the interactions (72.87% and 64.03%, respectively), with Proteobacteria dominating interactions in all four regions. At the genus level, *Stenotrophomonas* showed the highest degree of interactions in Rabat, *Erwinia* in Fes-Meknes and Doukkala, and *Lysinibacillus* in Abda.

#### 3.2.5. Core Microbiome Analysis of BSGM Populations

**Figure 6:**
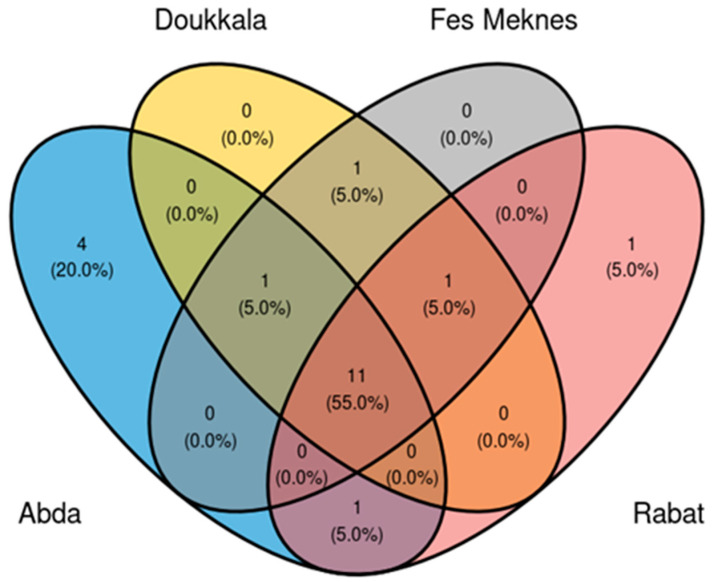
Venn diagram of the distribution of bacterial OTUs forming the core microbiome in *Mayetiola hordei* among the four examined regions.

The core bacterial community was composed of 20 OTUs out of 61 (33%). Among the four regions of Abda, Doukkala, Fes-Meknes, and Rabat, a core microbiome was detected, which included 11 OTUs (55%): *Stenotrophomonas* [OTU6], three variants of *Pseudomonas* [OTU2, OTU39, and OTU18], *Phyllobacterium* [OTU4], *Sphingomonas* [OTU26], *Ochrobactrum* [OTU30], *Chryseobacterium* [OTU21], *Delftia* [OTU14], *Candidatus* Nucleicultrix [OTU19], and *Novosphingobium* [OTU23]. Additionally, a core microbiome was observed exclusively in three regions: the case of two OTUs (10%): *Achromobacter* [OTU25] in Doukkala Rabat and Fes-Meknes, and *Cutibacterium* [OTU43] in Doukkala, Abda, and Fes-Meknes. Two OTUs (10%) were found in two regions: *Paracoccus* [OTU24] in Doukkala and Fes-Meknes, and *Cupriavidus* [OTU24] in Abda and Rabat. Finally, five OTUs (25%) were found only in one region, four in Abda (*Paracoccus* [OTU22], two variants of *Acinetobacter* [OTU12, OTU13], and *Brevundimonas* [OTU20]), plus one OTU from Rabat (*Bradyrhizobium* [OTU33]) (Figure 6) (see Table S7 for more details about the overall prevalence of the core microbiome).

## 4. Discussion

In the current study, we evaluated the prevalence of reproductive symbionts, the bacterial diversity of natural populations, and the impact of geographic origin on the bacterial community structure of barley stem gall midges, located in four different producing regions in Morocco. The bacterial microbiome analysis was performed using next-generation, high-throughput sequencing of the V3–V4 region of the bacterial 16S rRNA gene.

To analyze the existence of reproductive symbionts, a genus specific 16S rRNA PCR test was used. The screening results revealed that BSGM was only infected with *Wolbachia*. Surprisingly, *Wolbachia* infection was only found in BSGM populations from Rabat and Fez-Meknes, five larvae, two females, and three males from 44 samples from Rabat (22%), and one larva, one female, and one male from 52 individuals from Fez-Meknes (6%); in total 13 samples out of 152 BSGMs were infected, with a prevalence of 9%. However, none of the BSGM populations studied contained *Spiroplasma*, *Cardinium*, or *Arsenophonus*. The HTS DNA sequence datasets also revealed the presence of *Wolbachia* infections, which were detected in two out of four regions studied. Seven samples out of 20 were infected in Rabat (35%), while in Abda, one sample out of 18 (5%) was found to be infected with *Wolbachia* (Table S6). The discrepancy in prevalence between the two methods may be attributable to the low *Wolbachia* infection density, as well as to the exclusion of some samples from the HTS sequence datasets due to their low read counts (less than 1000) and the higher sensitivity of HTS compared to conventional PCR screening [54,59]. To the best of our knowledge, *Wolbachia* had never been identified in BSGM populations. However, it was detected in a recent study of Hessian fly originating from Morocco, with a total of 13 samples out of 244 (5%) carrying *Wolbachia* infections: three females and ten males. Concerning HTS DNA sequence datasets, *Wolbachia* infections were also detected: 31 individuals out of 40 (78%) in various Hessian fly populations [34]. The Hessian fly shares with BSGM a similar distribution, similar morphology, and comparable symptoms in host plants [29]. On the other hand, no *Wolbachia* was found in the few other Hessian fly studies [35,60,61]. *Wolbachia* was also reported in the Asian rice gall midge (*Orseolia oryzae*) in India, where the sequencing of the 16S rRNA bacterial gene (V3-V4 region) revealed that *Wolbachia* was the predominant bacterium in the pupae and adults of both genders, with prevalences of 97.3%, 89.8%, and 79.6%, respectively [62]. In addition, *Wolbachia* is the most widespread intracellular bacterial genus, infecting filarial nematodes and a wide range of arthropod groups, including almost 65% of insect species [63,64,65], such as aphids [36,66,67,68] and fruit flies (Diptera:Tephritidae) [69,70,71].

Our efforts to identify *Wolbachia* strains found in natural populations of BSGM were limited by the difficulty in amplifying MLST (multi-locus sequence typing) and *wsp* genes, which could be attributed to the low infection density of *Wolbachia* [72,73]. The phylogenetic analyses were limited to 16S rRNA sequences amplified using *Wolbachia*-specific primers and the *Wolbachia*-related sequences acquired using HTS. All *Wolbachia* sequences found in this investigation exhibited the highest homology to strains from supergroup A (Figure 1 and Figure 6), indicating that the provenance of the BSGM samples did not contribute to the diversification of *Wolbachia* strains. It was previously discovered that the density of *Wolbachia* in a host influences the level of CI [74]. In light of this, the low infection rate found in our study implies that *Wolbachia* is unlikely to cause CI in the studied BSGM populations. Additionally, the fact that both males and females carry *Wolbachia* may indicate that the infection should not have any consequences on the sex ratio in favor of females. Even though *Wolbachia* are typically transmitted vertically, the population of barley midges in Rabat, Fes-Meknes, and Abda most likely became infected through horizontal transmission, which is common among insects [75,76] and may be mediated by parasitoids or host plants [77,78]. However, our results provide a fresh insight into the BSGM capacity to harbor the *Wolbachia* infection that may allow us to use the transinfection method to establish a reliable *Wolbachia*-infected lineage, by directing symbiont strains to be transferred to new hosts either within the same species or between species. Since it has been previously accomplished, the case of *Wolbachia* transinfections includes transporting strains between *Drosophila* species [79,80], *Aedes aegypti* [81,82,83], *Culex quinquefasciatus* [84], *Bactrocera oleae* [85], and *Ceratitis capitata* [86,87,88,89].

In order to investigate the effect of geographical origin on bacterial structure, HT sequencing was applied on 75 natural barley stem gall midge samples, based on a 90% sequence similarity. The results reveal a high number of distinct OTUs from four separate BSGM populations (Table S5 and Figure 3c). Our findings demonstrate that native environments have a major impact on the microbiota. The four BSGM natural populations were distinguished by various levels of species richness and diversity. Most bacterial species were found in the samples from Abda, Doukkala, and Fes-Meknes. In contrast, Rabat samples had statistically lower species richness and diversity. Additionally, samples from the Doukkala region displayed significantly higher diversity than samples from the Rabat region, according to the Shannon index. Since the research on the bacterial microbiome of this insect is still limited, it is the first time that changes in the structure of microbial communities have been observed in populations of BSGM with various geographic distributions. However, it has also been reported in other fly or insect species, including cases of the Hessian fly *(M. destructor*) [34,61], oriental fruit fly (*Bactrocera dorsalis*) [90,91], and melon fly (*Zeugodacus cucurbitae*) [92,93].

Proteobacteria was the predominant phylum detected in the four regions of Abda, Doukkala, Fes-Meknes, and Rabat. This phylum was also the most abundant in other insects, such as the Hessian fly from Morocco (three out of four regions) [34], the Asian rice gall midge (*O. oryzae*) from India [63], and the melon fly from Bangladesh (*Z. cucurbitae*) [92,93]. However, the classification of the rest of the phyla, Actinobacteria, Bacteroidetes, and Firmicutes, was different from one region to the other, in agreement with the results for other insects [34,62,92]. Additionally, a recently characterized phylum was detected in our study as the fifth most-common phylum among the four studied regions, with a prevalence of 32% (24 out of 75 samples), that is, candidate phylum Dependentiae (also known as TM6). The identification of this phylogenetic group of bacteria was based on isolates infecting free-living amoebae and on metagenomic studies. This phylum is common in a variety of habitats, including hospital biofilms, soil, waste water, and peat bogs; it is characterized by a relatively limited metabolic potential, but encodes a vast array of transporters, including ATP transporters and genes with bacterial endosymbiont-enriched activities [94,95,96,97,98]. At the class level, Gammaproteobacteria and Alphaproteobacteria were the most prevalent members in all samples analyzed, followed by Bacilli in Abda, Doukkala, and Fes-Meknes, and Actinobacteria in Rabat. However, in the Hessian fly populations from Morocco, the most frequent classes were Gammaproteobacteria, Alphaproteobacteria, and Betaproteobacteria [34]. Interestingly, members of Negativicutes were also detected but at a much lesser degree (Figure 3b), with a prevalence of 9.33% (seven out of 75 samples). This class was recently certified in the phylum Firmicutes that has generally been defined by having a low genomic GC content, Gram-positive (monoderm) cells, and an envelope composed of a cytoplasmic membrane and a thick coating of peptidoglycan [99]. However, Negativicutes are diderms, characterized by an inner and outer membrane containing lipopolysaccharides [100,101]. In addition, Negativicutes have colonized a variety of hosts, including insects, animals, soil, ocean, and sediments. They mainly rely on fermentation because they cannot grow in the presence of oxygen. Some members of Negativicutes are known by their ability to ferment lactate, as in the case of *Veillonella*, the genus that was present in our results, while other Gram-negative members can produce endospores [102,103]. At the genus level, *Pseudomonas* exhibited the highest relative abundances in Abda, Doukkala, and Rabat; this bacterial genus is known from a wide range of habitats and hosts and exhibits remarkable metabolic diversity [104,105]. The genus has adapted to interact specifically in both useful and harmful ways, mostly with plants [106,107], humans [108], and insects [109,110]. In the same three regions, *Pseudomonas* was followed by *Stenotrophomonas*, a genus reported from a variety of habitats that is frequently characterized as a multidrug-resistant, opportunistic human pathogen [111]. However, they are more usually discovered in soils or in close proximity to plants [112], where they can create symbiotic relationships [113]. *Stenotrophomonas* is also linked to a variety of insect species [114,115]. On the other hand, *Pantoea* was prevalent in the region of Fes-Meknes; this genus has regularly been isolated from a variety of terrestrial and aquatic ecosystems, in addition to connections with humans, animals, and insects [116,117]. Additionally, *Pantoea* isolates have been used as immunopotentiators to create supportive medications for melanoma, infections, allergies, and the reversal of immunosuppression [118,119,120]. Furthermore, several *Pantoea* isolates have been reported to produce antimicrobials and have been converted into commercial biocontrol treatments [121,122,123]. *Pantoea* was followed by *Pseudomonas* in the region of Fes-Meknes; this genus was also present in Hessian flies with different frequencies among different populations [34]. The reported variation in the microbiome of BSGM populations could be influenced by barley cultivars, insect pressure, environmental factors, including annual mean temperature, precipitation, and maximum snowfall, as well as the effects of host plants and insect developmental stages and sexes, plus the effect of *Wolbachia* infection on the microbiome [124,125].

## 5. Conclusions

The bacterial composition of populations of BSGM was found to be significantly influenced by the geographic location. With the highest number of OTUs, Proteobacteria were found to be the most abundant phylum in flies from the four sites. To the best of our knowledge, this is the first study of the BSGM bacterial microbiome that reports *Wolbachia* infection in this insect. The low infection rate identified in our study led us to suspect that *Wolbachia* is unlikely to be the source of cytoplasmic incompatibility in BSGM reproduction. On the other hand, our findings may offer a new perspective on the application of *Wolbachia* in the control of BSGM.

## Figures and Tables

**Table 1:** Number of collected barley stem gall midge adults and larvae collected from different locations.

Region	Location	Coordinates				Number of Insects		
		Altitude	Latitude	Longitude	Temperature	Larvae	Male	Female
Abda	Ras El Ain	183	32.16898	−8.55100	23 °C	8	-	-
	Khatazakane	58	32.17595	−9.10573	12 °C	10	-	-
Rabat	Tiflet	333	33.53909	−6.21687	16 °C	10	-	-
	SidiAllal Bahraoui	166	34.00966	−6.33159	16 °C	-	11	11
	Marchouch	391	33.60060	−6.710450	17 °C	2	-	-
	Rommani	434	33.557.628	−6.583154	19 °C	10	-	-
Fes-Meknes	Iqaddar	565	33.58706	−5.35273	18 °C	10	-	-
	Majjate	706	33.780093	−5.490483	19 °C	20	11	11
Doukkala	Chaibate	99	33.01083	−8.29074	20 °C	28	-	-
	Oulad Hamdane	82	33.11217	−8.23345	21 °C	10	-	-

## Data Availability

All the data that support the findings of this study are available in NCBI under BioProject PRJNA915357.

## Supplementary Materials

The following supporting information can be downloaded at https://www.mdpi.com/article/10.3390/microorganisms11030797/s1, Table S1. Prevalence of bacterial endosymbionts screened in populations of barley stem gall midges. Table S2. List of bacterial primers and annealing temperatures used; Table S3. Distribution of Dependencia family (Otu 45); Table S4. Distribution of Negativicutes class (Otu 106); Table S5. Relative abundance (%) of different bacteria detected in Barley stem gall midge regions of Abda, Doukkala, Fes Meknes, and Rabat; File S1. *Wolbachia* sequence alignments; Figure S1. Pairwise non-metric multidimensional scaling (NMDS) plot of bacterial communities in barley stem gall midge samples collected from Rabat (purple), Fes-Meknes (blue), Abda (red), and Doukkala (green) (*p* < 0.001). The ’d’ stands for the grid’s dissimilarity scale (d = 0.2 implies that the distance between two grid lines represents approximately 20% dissimilarity between the regions); Figure S2. Species richness and diversity indices with significance differences in natural populations of barley stem gall midge samples collected from the regions of Rabat, Fes Meknes, Abda, and Doukkala. The interquartile range (IQR) is represented by boxes, the median is represented by a line within the boxes, and samples are represented by dots (* 0.01 < *p* < 0.05, ** *p* ≤ 0.01); Figure S3. Co-occurrence and mutual exclusion networks at the genus level for OTUs that compose the bacterial communities of *Mayetiola hordei* natural populations from the four regions of (a) Fes-Meknes, (b) Rabat, (c) Doukkala, and (d) Abda. The degree of interaction determines the size of each node. Cases of copresence are represented by green edges, while mutual exclusion is represented by red edges. Table S6. Distribution of *Wolbachia* genus (Otu 156). Table S7. Overall core prevalence.
