# Supplementary material for: Characterization of the Bacterial Microbiome in Natural Populations of Barley Stem Gall Midge, Mayetiola hordei, in Morocco
Source: Microorganisms. 2023 Mar 21;11(3):797. doi: 10.3390/microorganisms11030797 (PMC10051481; doi:10.3390/microorganisms11030797)
Supplement: Supplementary file 1 [file microorganisms-11-00797-s001.zip › Supplementary Materials.pdf]

Supplementary Materials

Table S1. Prevalence of bacterial endosymbionts screened in populations of barley stem gall midge.

Population	Gender	Sample Size	*Wolbachia*	*Spiroplasma*	*Cardinium*	*Arsenophonus*
Rabat	Larvae	22	+(5)	-	-	-
	Male	11	+(3)	-	-	-
	Female	11	+(2)	-	-	-
Fes-Meknes	Larvae	30	+(1)	-	-	-
	Male	11	+(1)	-	-	-
	Female	11	+(1)	-	-	-
Doukkala	Larvae	38	-	-	-	-

+ infected individuals (the number of infected samples per location), − uninfected individuals.

Table S2. List of bacterial primers and annealing temperatures used.

Gene	Primer	Sequence (5'-3')	Tm °C	Product size
Wolbachia	WspecF	YATACCTATTCGAAGGGATAG	54 °C	438 bp
	WspecR	AGCTTCGAGTGAAACCAATTC		
Spiroplasma	63F	GCCTAATACATGCAAGTCGAACGG	57 °C	450 bp
	TKSS	TAGCCGTGGCTTTCTGGTAA		
Cardinium	CloF1	GGAACCTTACCTGGGCTAGAATGTATT	55 °C	466 bp
	CloR1	GCCACTGTCTTCAAGCTCTACCAAC		
Arsenophonus	ArsF	GGGTTGTAAAGTACTTTCAGTCGT	56 °C	600 bp
	ArsR3	CCTYTATCTCTAAAGGMTTCGCTGGATG		
12S rRNA	12SCFR	GAGAGTGACGGGCGATATGT	54 °C	377 bp
	12SCRR	AAACCAGGATTAGATACCCTATTAT		
16S rRNA V3-V4 region	U341F	CCTACGGGRSGCAGCAG	53 °C	464 bp
	805R	GACTACCAGGGTATCTAAT		
16S rRNA	27F	AGAGTTTGATCCTGGCTCAG	54 °C	1500bp
	1429R	GGTTACCTTGTTACGACTT		

Table S3. Destribution of Dependencia family (Otu 45)

Region	Doukkala						Abda						Rabat					Fes-Meknes							Total
Sample code	I13	I14	I15	I17	I4	I8	I24	I29	I31	I32	I33	I34	I40	I41	I44	I46	I47	I59	I61	I63	I67	I69	I73	I76	24
Reads	285	99	134	33	382	429	429	38	143	125	703	45	32	37	228	101	122	922	296	331	25	73	148	42	5202

Table S4. Destribution of Negativicutes class (Otu 106)

Region	Doukkala					Abda	Fes-Meknes	Total
**Sample code**	**I7**	**I9**	**I16**	**I17**	**I18**	**I23**	**I67**	**7**
**Reads**	**214**	**300**	**1190**	**933**	**534**	**933**	**150**	**4254**

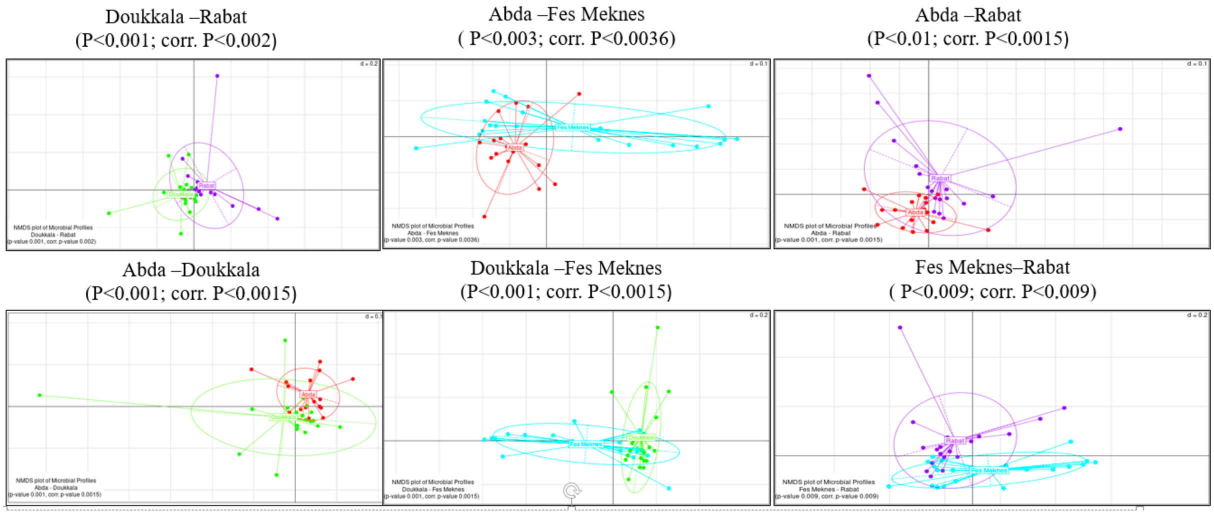

Figure S1. Pairwise non-metric multidimensional scaling (NMDS) plot of bacterial communities in Barley stem gall midge samples collected from Rabat (purple), Fes-Meknes (blue), Abda (red), and Doukkala (green) ($p < 0.001$). The'd' stands for the grid's dissimilarity scale (d = 0.2 implies that the distance between two grid lines represents approximately 20% dissimilarity between the regions).

Table S6: Destribution of Wolbachia genus (Otu 156)

Region	Abda	Rabat						Total
**Sample code**	**I22**	**I41**	**I43**	**I49**	**I50**	**I54**	**I55**	**I56**	**8**
**Reads**	**4**	**7**	**10**	**11**	**451**	**125**	**260**	**10**	**878**

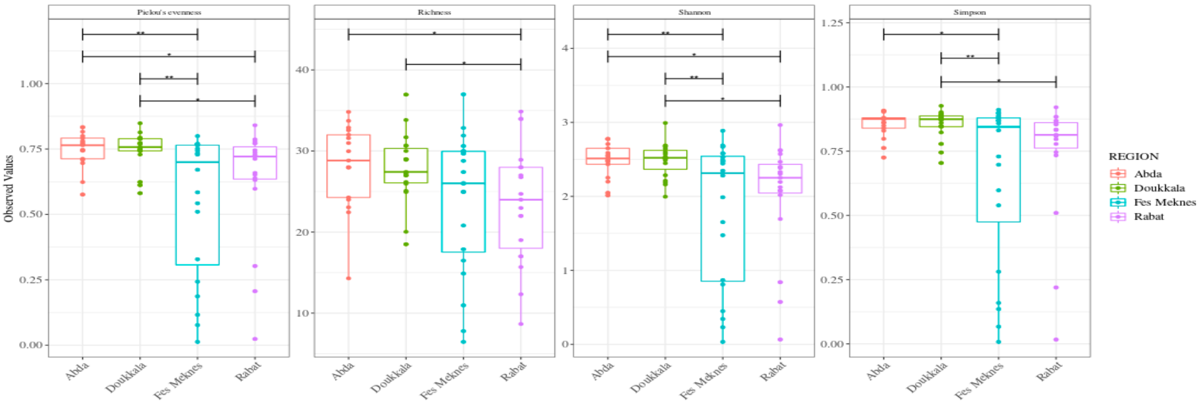

Figure S2. Species richness and diversity indices with significant differences in natural populations of Barley steam gall midge samples collected from the regions of Rabat, Fes Meknes, Abda, and Doukkala. The interquartile range (IQR) is shown by boxes, a line within the boxes represents the median, and samples are represented by dots. (* $0.01 < p < 0.05$, ** $p \leq 0.01$).

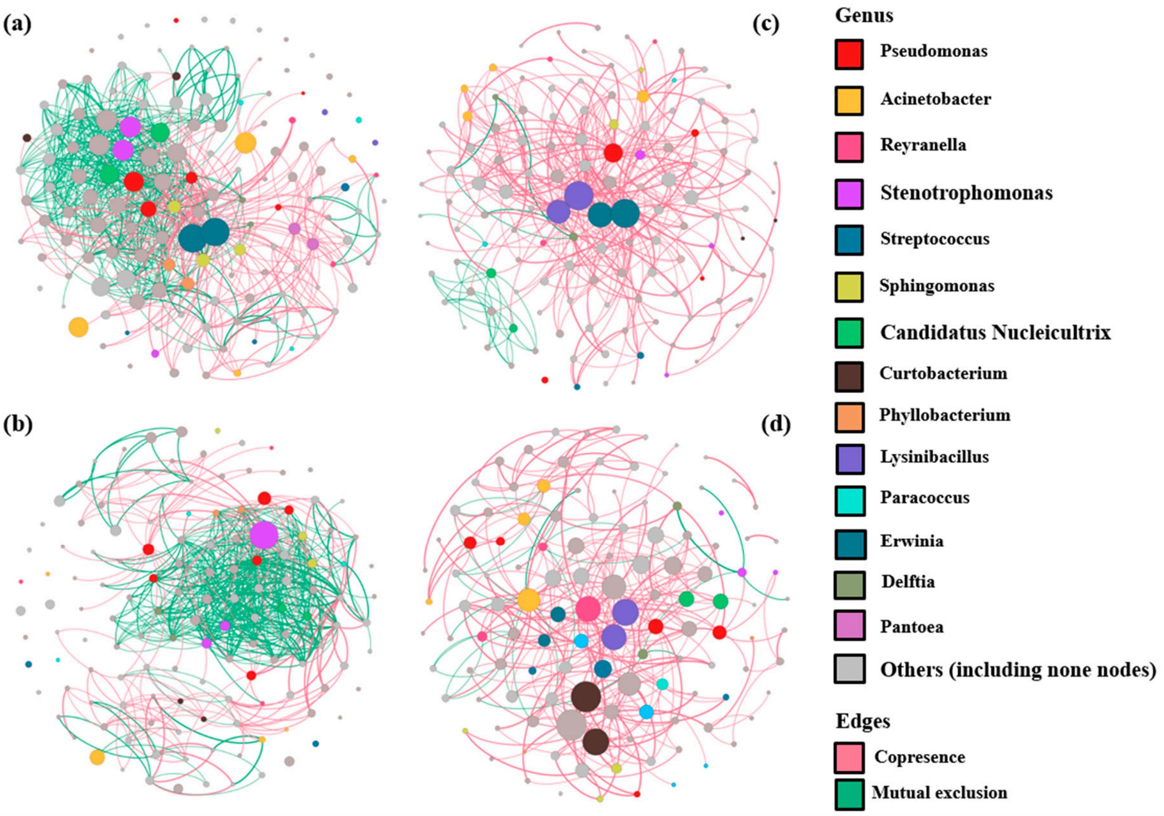

Figure S3. Co-occurrence and mutual exclusion networks at the genus level for the OTUs that compose the bacterial communities of *Mayetiola hordei* natural populations from the four regions, (a) Fes-Meknes, (b) Rabat, (c) Doukkala, and (d) Abda. The degree of interaction determines the size of each node. Cases of copresence are shown by green edges, while mutual exclusion is represented by red edges.
